# Supplementary material for: Drug-tunable multidimensional synthetic gene control using inducible degron-tagged dCas9 effectors
Source: Nat Commun. 2017 Oct 30;8:1191. doi: 10.1038/s41467-017-01222-y (PMC5662744; doi:10.1038/s41467-017-01222-y)
Supplement: Supplementary file 1 — Supplementary Information [file 41467_2017_1222_MOESM1_ESM.pdf]

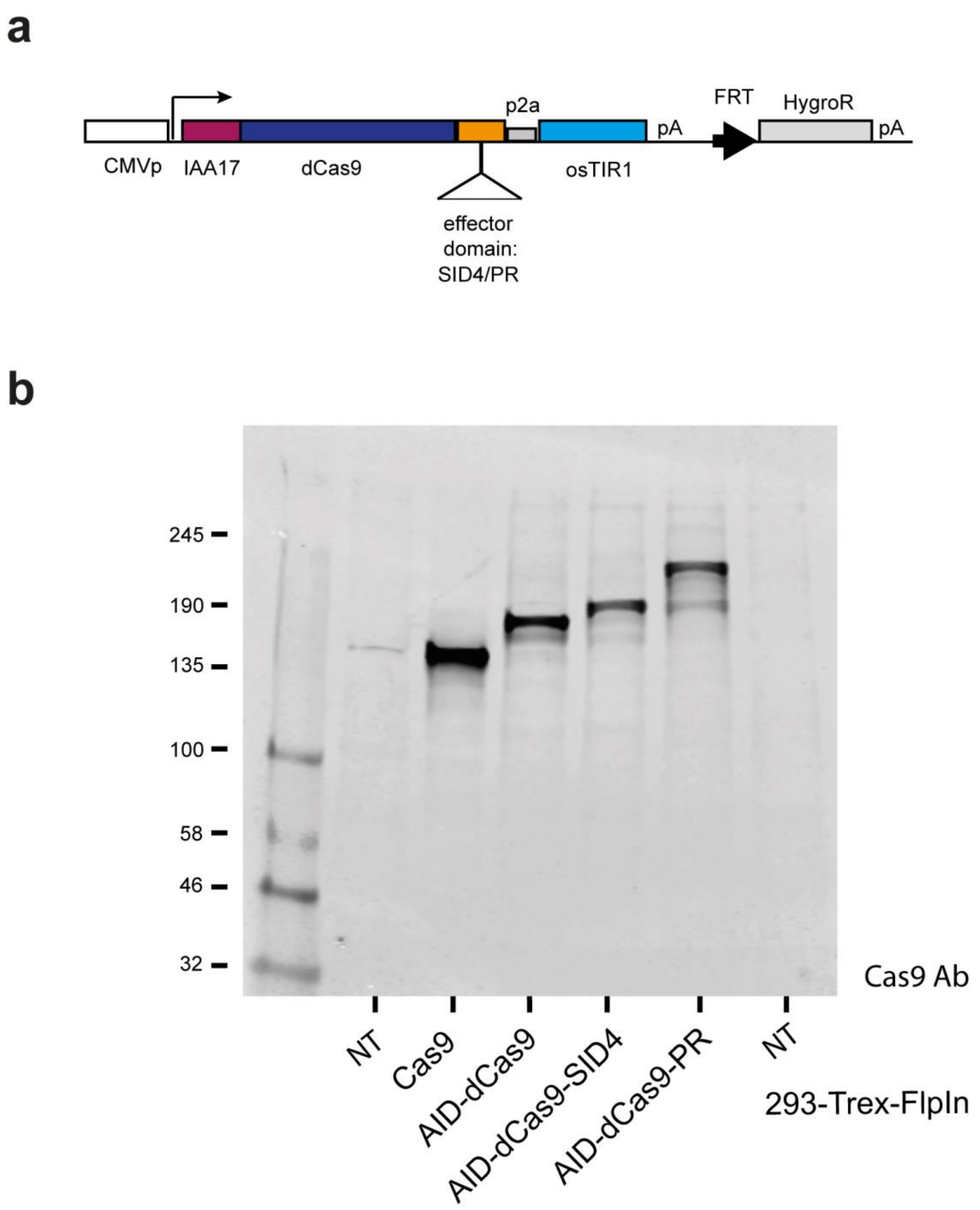

Supplementary Figure 1: Attachment of various transcriptional effector domains to AID-dCas9. (**a**) Schematic representation of the AID-dCas9-effector construct. Expression is driven from a CMV promoter. The IAA17 degron is cloned at the N-terminal side of nuclease-dead Cas9 (D10A, H840A), with various effector domains attached at the C-terminus. Co-expression of the osTIR1 gene is achieved through linkage via a p2a peptide bridge. A Flp recombinase site followed by a promoter-less hygromycin resistance gene is included to allow Flp-ase mediated stable integration into FRT containing genomic landing pad sites. (**b**) Western blot of HEK293-Trex-FlpIn clones stably expressing AID-dCas9 variants without effector domain (lane 3), with a 4 copy module of the Sin3a interacting domain (SID4) (lane 4) or a p65-AD-rTa transactivation domain (lane 5). Samples of non-transfected cells (lanes 1 and 6) and cells transiently transfected with Cas9 (lane 2) are included for comparison.

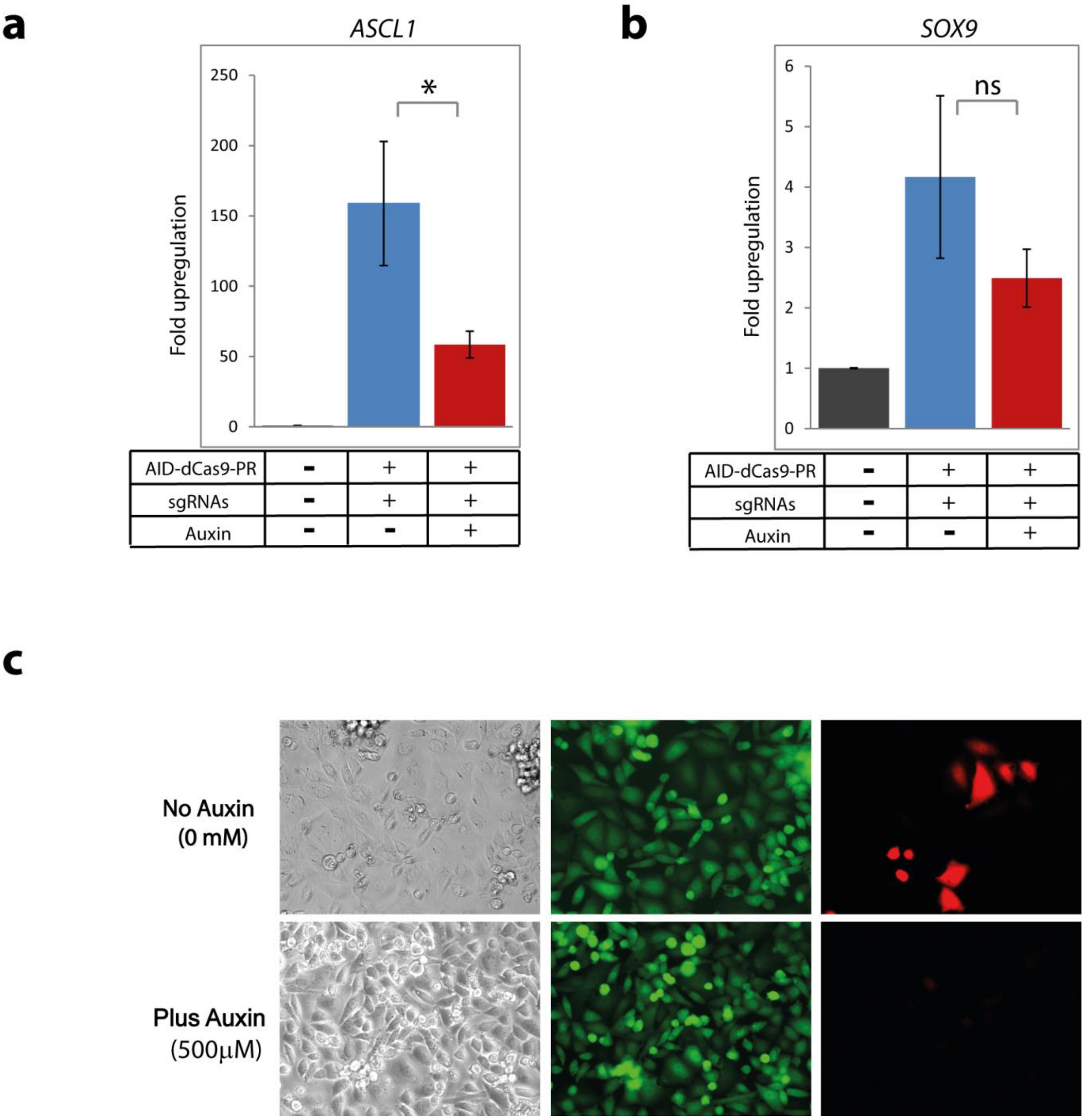

Supplementary Figure 2: Auxin dependent induction of gene expression by AID-dCas9-PR. (**a,b**) Upregulation of endogenous genes in HEK293FT cells transiently transfected with AID-dCas9-PR is dependent on the addition of auxin to the media. rt-qPCR analysis of the *ASCL1* (a) and *SOX9* (b) genes in the presence (lanes 2) or absence (lanes 3) of 500 μM auxin. Error bars denote ± s.d. (n=3) (2-tailed t-test, (**) P<0.01). (**c**) Induction of mKate reporter gene expression in CHO cells with AID-dCas9-PR stably integrated into a constitutive GFP-expressing landing-pad site upon transfection with a guideRNA binding to eight sites in its promoter is abrogated in the presence of auxin in the culture media.

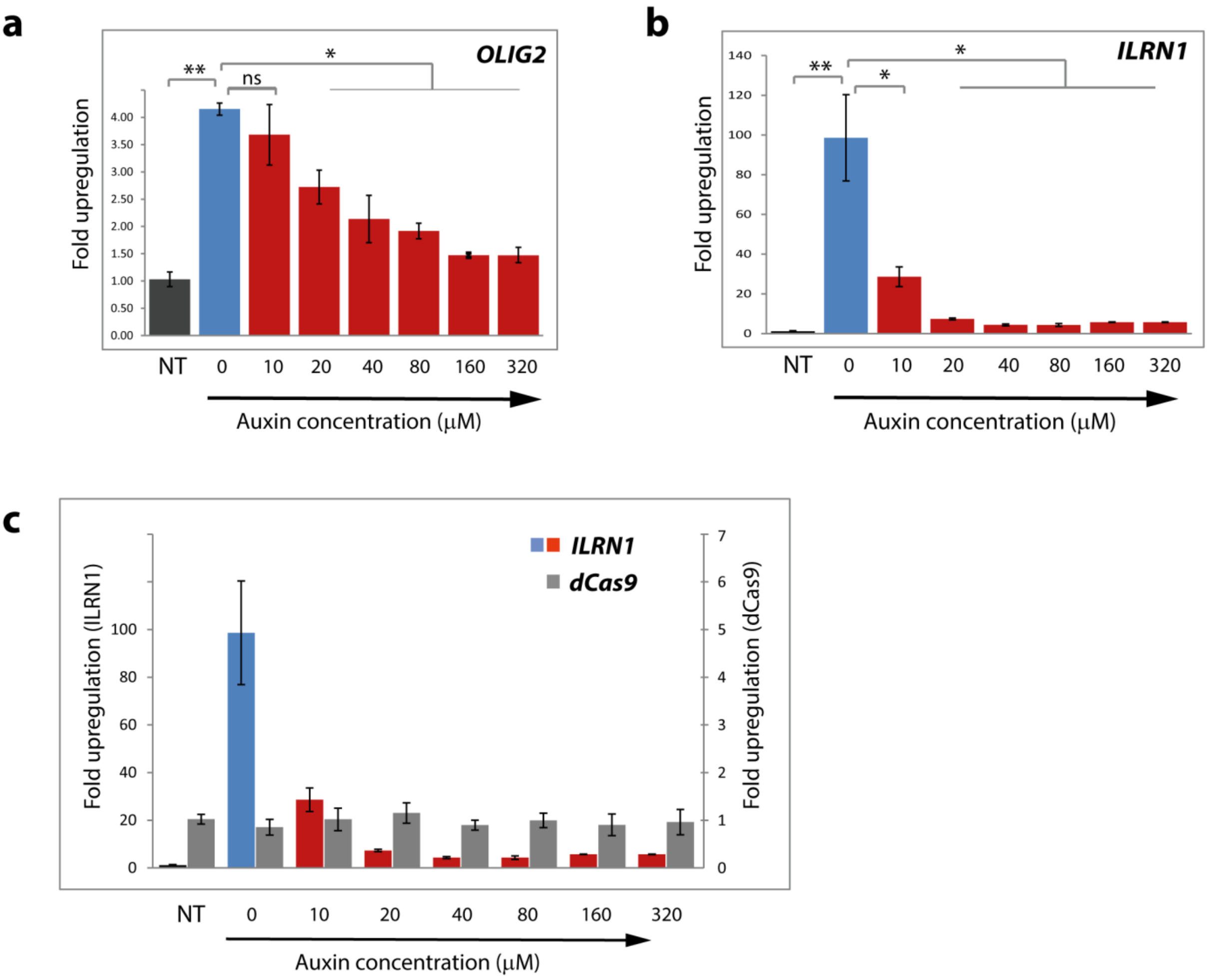

Supplementary Figure 3: Tunability of functional activity of AID-dCas9 effectors. A stable AID-dCas9-PR expressing cell line was transfected with a mix of guideRNAs against multiple sites within the promoter regions of the *OLIG2* (a) or *IL1RN* target (b) regions. Auxin was added to the concentrations indicated. mRNA induction levels decreased with increasing auxin concentration. NT, no guideRNA transfected. Error bars denote ± s.d. (n=3), (2-tailed t-test, (**) P<0.01, (*) P<0.05, (ns) not significant). (c) mRNA levels of dCas9-PR itself remained constant under different auxin concentrations, showing that auxin addition affects the level of dCas9-effectors at the protein level.

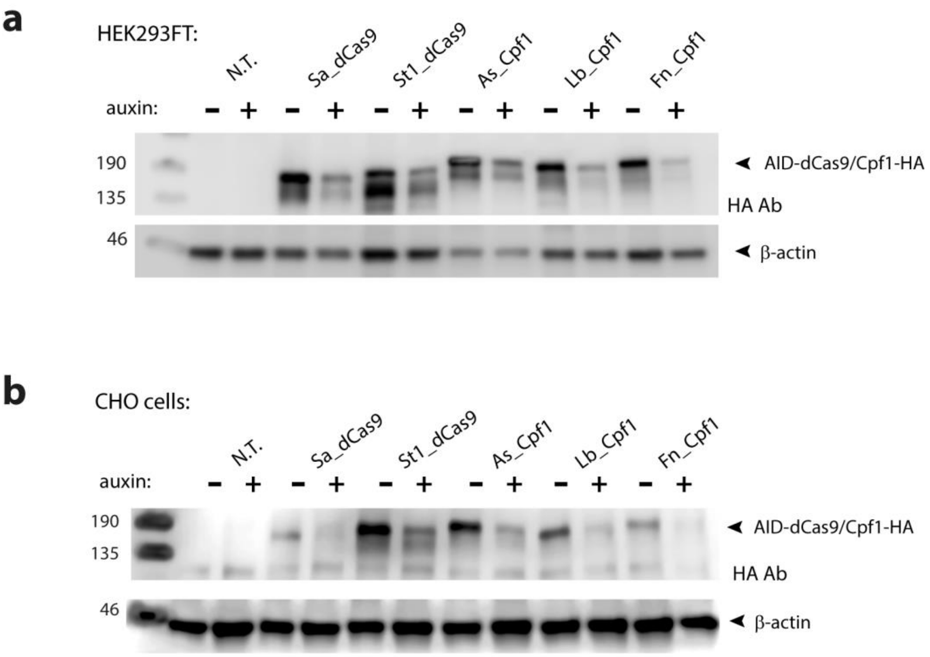

Supplementary Figure 4: Stability of orthogonal dCas9 and Cpf1 proteins fused with the AID. (**a,b**) Western blots of degron-tagged dCas9/Cpf1 fusion proteins expressed from AID-cDNA-HA-p2a-osTIR1 all-in-one constructs in the presence or absence of added auxin in HEK293FT (a) or CHO-K1 (b) cells. Sa_dCas9, Staph Aureus dCas9, St1_dCas9, Streptomyces thermophilus 1 dCas9, As_Cpf1, Acidaminococcus sp BV3L6 Cpf1, Lb_Cpf1, Lachnospiraceae bacterium ND2006 Cpf1, Fn_Cpf1, Francisella novicida Cpf1.

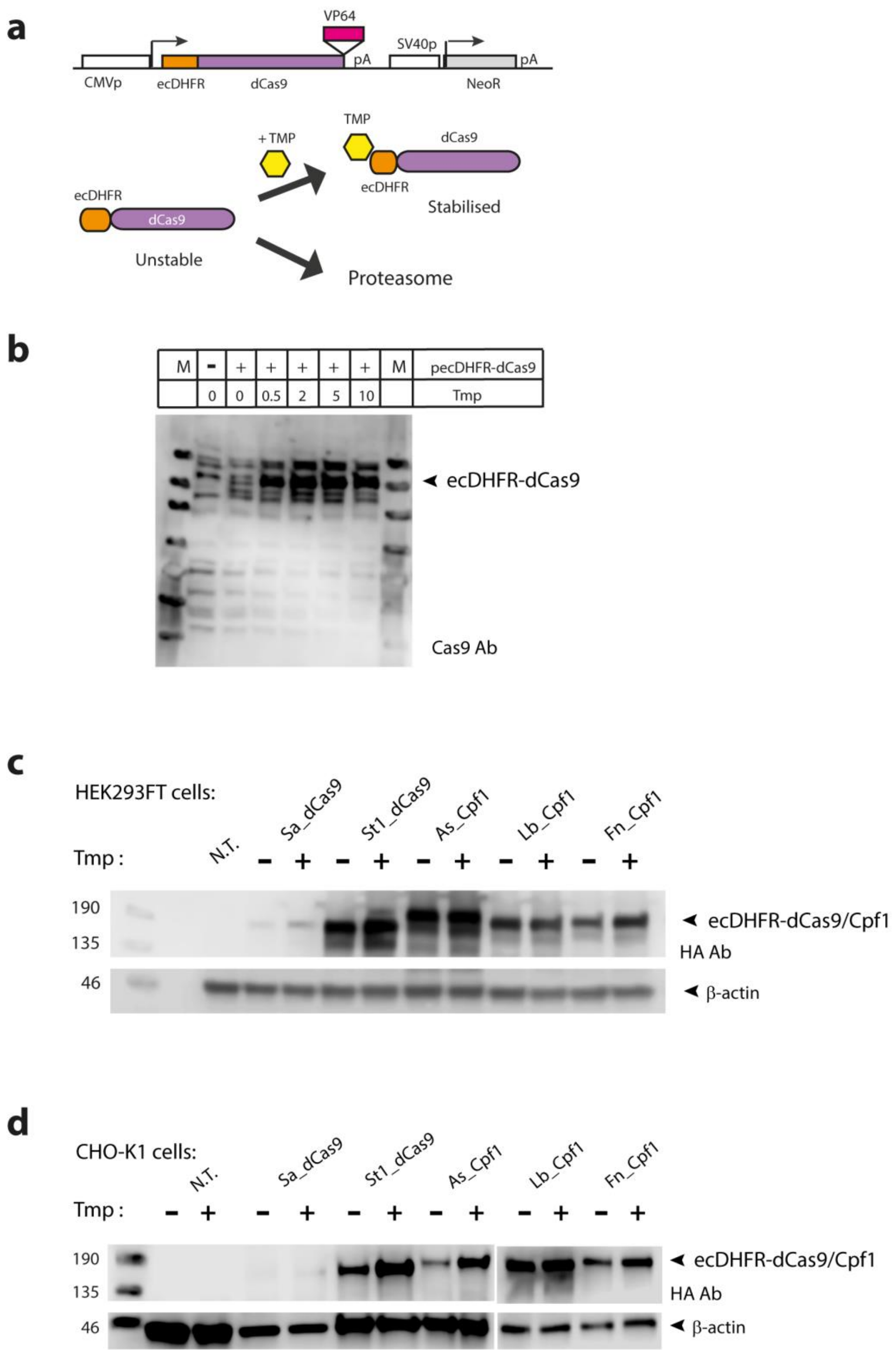

Supplementary Figure 5: Stability of orthogonal dCas9 and Cpf1 proteins fused with the ecDHFR degron. (**a**) Schematic overview of the ecDHFR degron system. Tagging with the ecDHFR destabilisation domain confers instability to resulting fusion proteins leading to degradation by the proteasome. Addition of TMP stabilises the fusion product. (**b**) N-terminal fusion of the ecDHFR DD creates a destabilised S.pyogenes dCas9, which is stabilised by addition of TMP in a concentration-dependent manner. (**c,d**) Application of the ecDHFR degron to the set of orthogonal dCas9 and Cpf1 proteins. Transfected cells with N-terminal ecDHFR fusion proteins from the species indicated were treated with TMP or vehicle and their stability assessed on Western blot with an antibody against their C-terminal HA tag in HEK293 cells (c) and CHO-K1 cells (d).

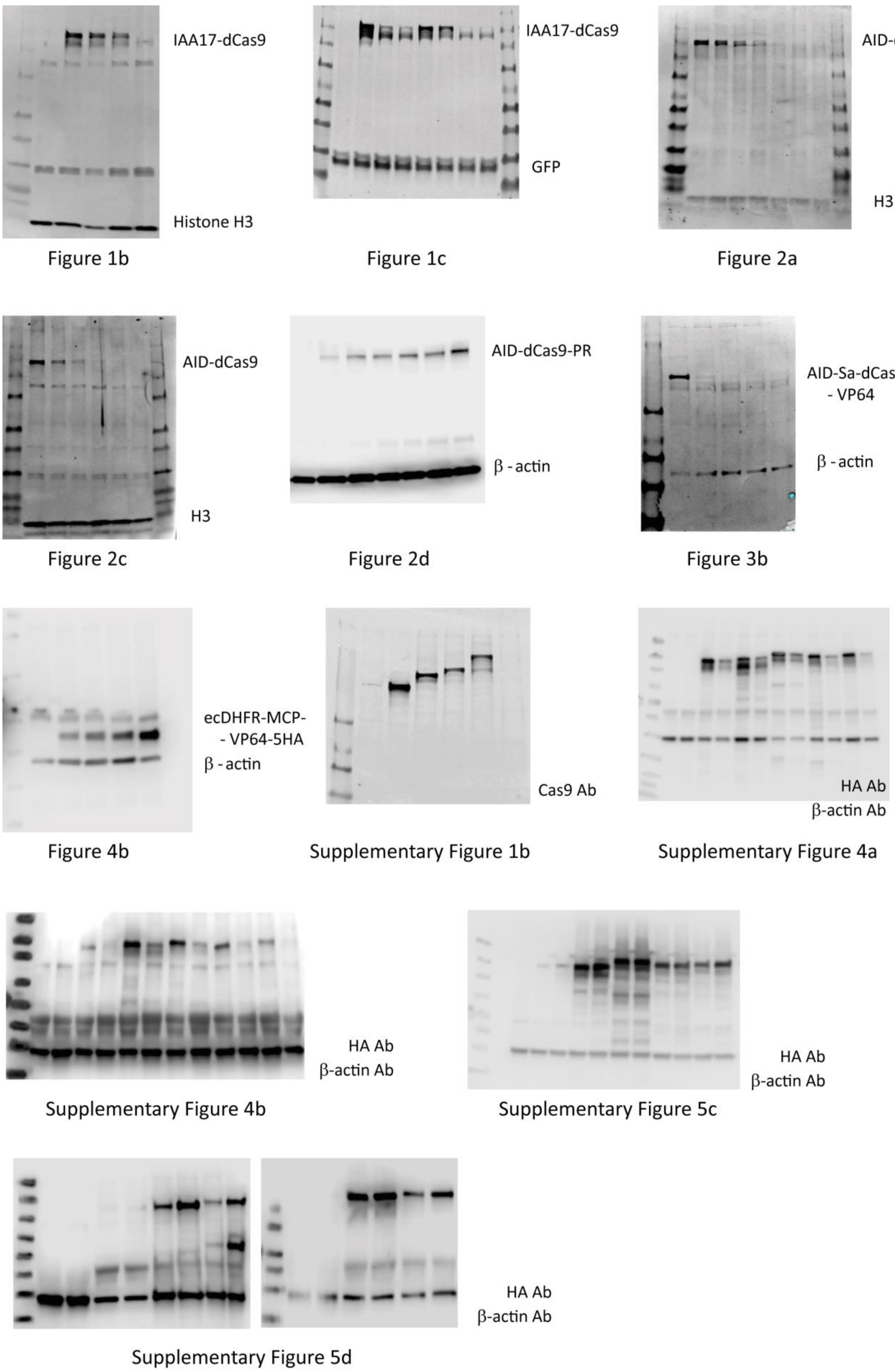

Supplementary Figure 6: Full images of Western blots used in the manuscript.

Supplementary Table 1: Single guideRNA and rtPCR primer sequences.

**Single guideRNA sequences:**		
**HEK293 sgRNAs:**		
	Target sequences (5' - 3'):	
SOX9 sgRNA_1	TACGACAAACTTACACACT	
SOX9 sgRNA_2	ACTGCTGTGCTGTGATTGG	
SOX9 sgRNA_3	ATTATTACGGAGGAACAGC	
SOX9 sgRNA_4	CAAAGCTAAGTCCCCGCGA	
IL1RN sgRNA1	TGTACTCTCTGAGGTGCTC	
IL1RN sgRNA2	ACGCAGATAAGAACCAGTT	
IL1RN sgRNA3	CATCAAGTCAGCCATCAGC	
IL1RN sgRNA4	GAGTCACCCTCCTGGAAAC	
ASCL1 sgRNA1	GCTGGGTGTCCCATTGAAA	
ASCL1 sgRNA2	CAGCCGCTCGCTGCAGCAG	
ASCL1 sgRNA3	TGGAGAGTTTGCAAGGAGC	
ASCL1 sgRNA4	GTTTATTCAGCCGGGAGTC	
OLIG2 sgRNA1	GCCTGGCGAGTTTCATTGAG	
OLIG2 sgRNA2	GACGGCGGCAGCGGTGGCGG	
OLIG2 sgRNA3	GCCACCACAGAGTCAGGTTG	
**CHO cell sgRNAs:**		
RasL11A sgRNA	GCGCCGCTCGGCGACAGCAG	
Arpc1b sgRNA	GAGTGCTCACTTCTGCAAGG	
**sgRNAs for artificial reporter gene promoters:**		
S.p_gRNA_1	GAGTCGCGTGTAGCGAAGCA	
S.a_gRNA_2	GCCTGAAAATATTAAGTAAGT	
**rt-qPCR primers:**		
**HEK293 cell rt-qPCR primers:**		
	forward (5'-3')	reverse (5'-3')
IL1RN rtPCR	GGAATCCATGGAGGGAAGAT	TGTTCTCGCTCAGGTCAGTG
SOX9 rtPCR	GAGGAAGTCGGTGAAGAACGG	TCGAAGGTCTCGATGTTGGAG
ASCL1 rtPCR	GGAGCTTCTCGACTTCACCA	AACGCCACTGACAAGAAAGC
OLIG2 qPCR	GGTAAGTGCGCAATGCTAAGCTGT	TACAAAGCCCAGTTTGCAACGCAG
RCN1 qPCR	CCCGTAATTGGTAGCTTGGC	TGAGATTCGCCACTGGATCC
GAPDH rtPCR	CCCATCACCATCTTCCAG	ATGACCTTGCCCACAGCC
dCas9 rtPCR	CGCAAACGCCCTCTAATCGA	CCGCCTGTCTGTACTTCTGT

CHO cell rt-qPCR primers:		
	forward (5'-3')	reverse (5'-3')
RasL11A qPCR	CTCGTCTATGTGGAGGGGGA	GGCAGAGGGGGCTTTTACTT
Arpc1b qPCR	TACACCGTGGGGCTCTAAGA	GGTCCTTCAAGGCTGACTCC
b-actin qPCR	CCACCATGTACCCAGGCATT	CGGACTCATCGTACTCCTGC
